# Clinical efficacy of Duyiwei capsule in treating gingivitis: A protocol of systematic review and meta-analysis

**DOI:** 10.1097/MD.0000000000020542

**Published:** 2020-07-17

**Authors:** Hai-Ying Liu, Jing-Hui Fan, Na Lin, Zhi-Xuan Zhao

**Affiliations:** a Department of Geriatrics and Integrated Traditional Chinese and Western Medicine; b Department of Pharmacy; c Department of Stomatology, Affiliated Hongqi Hospital of Mudanjiang Medical University, Mudanjiang, China.

**Keywords:** Duyiwei capsule, efficacy, Gingivitis, safety

## Abstract

**Background:**

This study will investigate the clinical efficacy of Duyiwei capsule (DYWC) for the treatment of gingivitis.

**Methods:**

Relevant studies will be searched in PUBMED, EMBASE, Cochrane Library, WANGFANG, VIP, CBM, and CNKI from inception to the March 31, 2020 without limitations of language and publication time. All potential randomized controlled trials on the clinical efficacy of DYWC for the treatment of gingivitis will be considered. Two authors will independently perform literature selection, data collection, and study quality assessment. Any disagreements will be solved by a third author through discussion. We will utilize RevMan 5.3 software for statistical analysis.

**Results:**

This study will summarize present randomized controlled trials on the efficacy and safety of DYWC for the treatment of gingivitis.

**Conclusion:**

The findings of this study will provide evidence to show whether DYWC is effective and safety for gingivitis.

**Systematic review registration:** INPLASY202040199.

## 1 Introduction

Gingivitis is a common periodontal disease,^[1–3]^ which may affect about 55.7% in general population.^[4]^ It is characterized by gingiva changes without loss of periodontal attachment.^[5–7]^ Patients with this condition often experience bleeding on tooth brushing, gingival swelling, and redness.^[8,9]^ Although several managements are reported that can benefit gingivitis, the efficacy is limited.^[10–12]^

Traditional Chinese medicine is widely used to treat a variety of diseases in China during the past time and present, and it has achieved satisfied efficacy. Recent studies reported that Duyiwei capsule (DYWC) can be utilized for the treatment of gingivitis.^[13–20]^ However, no systematic review was conducted in the past. Thus, this study aims to assess the efficacy and safety of DYWC for the treatment of gingivitis.

## 2 Methods

### 2.1 Study registration

This study has been registered on INPLASY202040199. It will be carried out under the guideline of Preferred Reporting Items for Systematic Reviews and Meta-analyses Protocols.^[21]^

### 2.2 Inclusion criteria for study selection

#### 2.2.1 Type of study

Only randomized controlled trials that assessed the efficacy and safety of DYWC for gingivitis will be included. We will exclude any other studies, such as animal studies, reviews, and nonclinical studies.

#### 2.2.2 Type of patients

The patients, over 18 years, who were diagnosed with gingivitis will be included in this study. There are no limitations related to the race, gender, and nationality.

#### 2.2.3 Type of interventions

Experimental interventions: All patients received DYWC as an interventional management, irrespective dosage, duration, and frequency.

Control interventions: All patients underwent any modalities as a comparison. However, we will exclude studies if they included studies involved any forms of DYWC.

#### 2.2.4 Types of outcome measurements

The primary outcomes are gingival index, dental plaque index, and bleeding on probing.

The secondary outcomes are gingival abrasion scores, bleeding scores, and adverse events.

### 2.3 Search methods for identification of studies

#### 2.3.1 Electronic databases

This study will search relevant studies in PUBMED, EMBASE, Cochrane Library, WANGFANG, VIP, CBM, and CNKI from inception to the March 31, 2020. There are no restrictions related to the language and publication time. We will include randomized controlled trials that assessed the efficacy of DYWC for the treatment of gingivitis. We will present detailed search strategy for PUBMED in Table 1. We also adapt similar detailed search strategy for other electronic databases.

**Table 1:**
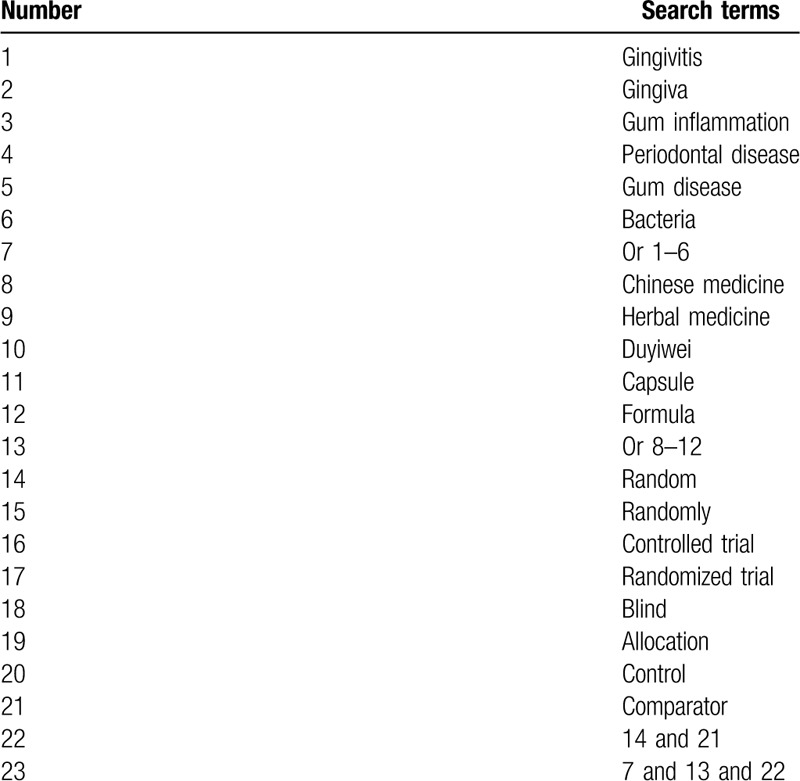
Search strategy for PUBMED.

#### 2.3.2 Searching other resources

Other resources of relevant studies will be identified, including websites of clinical trial registry, dissertations, conference papers, and reference lists of relevant literatures.

### 2.4 Data collection and analysis

#### 2.4.1 Study selection

Two authors will independently examine the titles and abstracts of all retrieved literatures, and will exclude all duplicated and irreverent studies. Then, full papers of potential studies are carefully read against all inclusion criteria. We will list reasons of all excluded studies and will present them in a table. If conflicts occur between 2 authors, a third author will be invited to solve them by discussion. The process of study selection is showed in a Preferred Reporting Items for Systematic Reviews and Meta-analyses Protocols flow chart.

#### 2.4.2 Data extraction and management

Two authors will independently extract data using a standardized data extraction form. If there are different views between 2 authors, we will invite a third author to settle them down through consultation. The extracted data includes publication information (eg, title, first author, and publication year), patients’ characteristics (eg, age, gender, and eligibility criteria), trial setting, trial methods, details of treatment and comparison (eg, delivery methods, dosage, and frequency), primary and secondary outcomes, results, findings, and other essential information. If unclear or missing data is examined, we will contact primary authors to achieve it.

#### 2.4.3 Assessment of risk of bias

Two authors will independently examine the risk of bias for all trials using Cochrane risk of bias. We will check it on 7 different levels, and will divide it into 3 degrees: low risk of bias, unclear risk of bias, and high risk of bias. If we identify any disagreements between 2 authors, we will resolve them by discussion with the help of a third author.

#### 2.4.4 Detection of treatment effect

We will detect treatment effect of dichotomous data as risk ratio and 95% confidence intervals, and will estimate treatment effect of continuous data as mean difference or standardized mean difference and 95% confidence intervals.

#### 2.4.5 Assessment of heterogeneity

Statistical heterogeneity will be identified by *I*^2^ statistics. *I*^2^ ≤ 50% means minor heterogeneity, and a fixed-effects model will be implemented, while *I*^2^ > 50% suggests major heterogeneity, and a random-effects model will be utilized.

#### 2.4.6 Subgroup analysis

A subgroup analysis will be conducted to explore the major heterogeneity based on the variations in study and patient characteristics, treatments, controls, and outcomes.

#### 2.4.7 Sensitivity analysis

A sensitivity analysis will be performed to examine the satiability of study findings by eliminating low quality studies.

#### 2.4.8 Publication bias

If there are adequate studies (normally over 10 trials), we will investigate the publication bias using Funnel plot and Egger regression test.^[22]^

#### 2.4.9 Data synthesis

This study will analyze data using RevMan 5.3 software. If minor heterogeneity is identified across 2 or more eligible studies on same outcomes, meta-analysis will be carried out based on the sufficient similarity in study and patient characteristics, details of interventions and controls, and outcome measurements. Otherwise, if major heterogeneity is investigated, a subgroup analysis and meta-regression test will be undertaken to explore the possible reasons of obvious heterogeneity. In addition, a narrative description will be used to report study results.

### 2.5 Dissemination and ethics

This study will not require ethical document, because this study will not use individual data. This study is expected to be published in a peer-reviewed journal.

## 3 Discussion

A number of previous studies have reported the clinical efficacy of DYWC for the treatment of gingivitis.^[13–20]^ However, there is no similar systematic review exploring this subject. This systematic review will be conducted to critically examine the literature on the treatment of gingivitis. Specifically, we will assess the efficacy and safety of DYWC for gingivitis. Findings from this study may supply the generation of clinical recommendations for clinician treating patients with gingivitis. Its results may also help inform future research in this field.

## Author contributions

**Conceptualization:** Hai-ying Liu, Zhi-xuan Zhao.

**Data curation:** Zhi-xuan Zhao.

**Formal analysis:** Hai-ying Liu.

**Investigation:** Zhi-xuan Zhao.

**Methodology:** Hai-ying Liu.

**Project administration:** Zhi-xuan Zhao.

**Resources:** Hai-ying Liu, Na Lin.

**Software:** Hai-ying Liu, Na Lin.

**Supervision:** Zhi-xuan Zhao.

**Validation:** Hai-ying Liu, Zhi-xuan Zhao.

**Visualization:** Hai-ying Liu, Na Lin, Zhi-xuan Zhao.

**Writing – original draft:** Hai-ying Liu, Na Lin, Zhi-xuan Zhao.

**Writing – review & editing:** Hai-ying Liu, Na Lin, Zhi-xuan Zhao.
